# Primary central nervous system lymphoma: A diagnostic challenge in a young immunocompetent patient with limited resources

**DOI:** 10.1016/j.radcr.2024.07.039

**Published:** 2024-08-07

**Authors:** Zekarias Seifu Ayalew, Mahlet Gebregiorgis, Gebeyehu Tessema Azibte, Abdurrhman Kedir Hamza, Isa Salo Abdo, Bereket Abraha Molla

**Affiliations:** a Department of Internal Medicine, Addis Ababa University, Addis Ababa, Ethiopia; b Division of Hematology, Department of Internal Medicine, Addis Ababa University, Addis Ababa, Ethiopia; c Department of Pathology, Addis Ababa University, Addis Ababa, Ethiopia

**Keywords:** PCNSL, Extranodal NHL, Immunocompetent, HIV

## Abstract

Primary central nervous system lymphoma is a rare form of central nervous system malignancy. It predominantly affects immunocompromised individuals and the elderly population. Diffuse large B-cell lymphoma is the most common type. This case report presents a 35-year-old female patient presented with progressive difficulty maintaining balance, headaches, seizures, and blurry vision for 2 months. Physical examination was unremarkable except for sluggish bilateral pupillary reaction and lower extremity weakness. MRI revealed multiple bilateral intraaxial masses. Biopsy and immunohistochemistry confirmed diffuse large B-cell lymphoma, nongerminal center B-cell type. However, the diagnosis was delayed for 4 months. The delay in the diagnosis was caused by its atypical presentation, a surgical site infection, and limited resources, which led the patient to disregard the recommended treatment and leave the hospital against medical advice. Even in the absence of risk factors of primary central nervous system lymphoma, it should be considered as a differential in a young patient with neurologic symptoms and intraaxial mass. Minimally invasive biopsy techniques and readily available immunohistochemistry are essential for prompt diagnosis and guiding treatment.

## Introduction

Non-Hodgkin lymphoma (NHL) can affect the central nervous system (CNS) in 2 ways: either through primary involvement of the CNS or through secondary dissemination, where it spreads from an aggressive NHL located elsewhere in the body. Primary CNS lymphoma (PCNSL) is a specific type of NHL that is confined to the CNS and accounts for approximately 1%-3% of all brain tumors [1,2]. PCNSL is an extranodal NHL that comprises 4%-6% of all NHL [3] The two important risk factors for PCNSL are immunosuppression and age. The median age at diagnosis is 60 years [1,4]. PCNSL is more likely to be in younger, female sex, and multifocal in immunocompromised patients than in immunocompetent patients [5]. High-dose intravenous methotrexate is an effective treatment for PCNSL. It is often combined with other chemotherapy agents or radiotherapy to achieve optimal outcomes [6]. Initial corticosteroid therapy can be used to alleviate neurological symptoms associated with PCNSL. It may also have some prognostic value [7]. Corticosteroid use could affect the pathological diagnosis of PCNSL, but this is not the case for the majority of patients [8]. We presented a young immunocompetent patient diagnosed with PCNSL with multifocal brain involvement.

## Case presentation

A 35-year-old female patient presented with progressive difficulty maintaining balance, which began while walking and deteriorated to the point where she was unable to ambulate independently over the past 2 months. She also reported dull, constant global headaches for the past 2 months, 2 episodes of generalized tonic-clonic seizures within the last month, and blurry vision that began 1 month ago and has worsened progressively over the past 2 weeks, accompanied by projectile vomiting. Otherwise, there was no history of difficulty with speech, sensory loss, or urinary incontinence. She denied any fevers, cough, or known tuberculosis exposure. She reported no weight loss or night sweating. Her medical history was unremarkable for diabetes mellitus, hypertension, malignancy (personal or familial), solid organ transplantation, HIV infection, immunosuppressant use, or autoimmune disease.

Vital signs were stable, and general physical examinations were unremarkable. The GCS was 15/15. Pupils were midsize and sluggish in response to light. Visual acuity testing revealed no light perception bilaterally. On motor examination, power was 4/5 on both lower extremities and reflexes were brisk. Examination of the cranial nerves showed no abnormalities. No sensory deficit was identified.

**Fig. 1:**
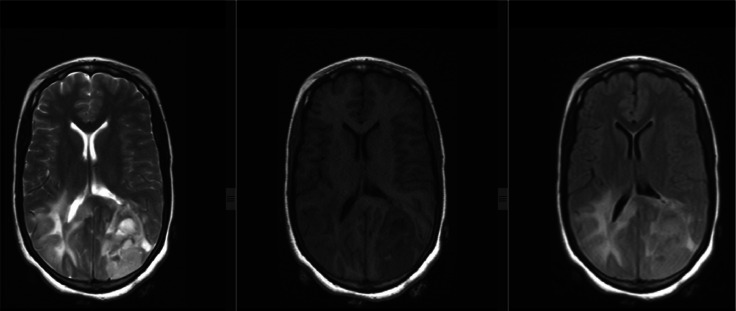
MRI images of PCNSL. From left to right, T2 hyperintense, T1 hypointense, and FLAIR hyperintense subcortical and cortical lesions involving the bilateral occipito-parietal lobe, temporal lobe, and parietal lobes.

**Fig 2:**
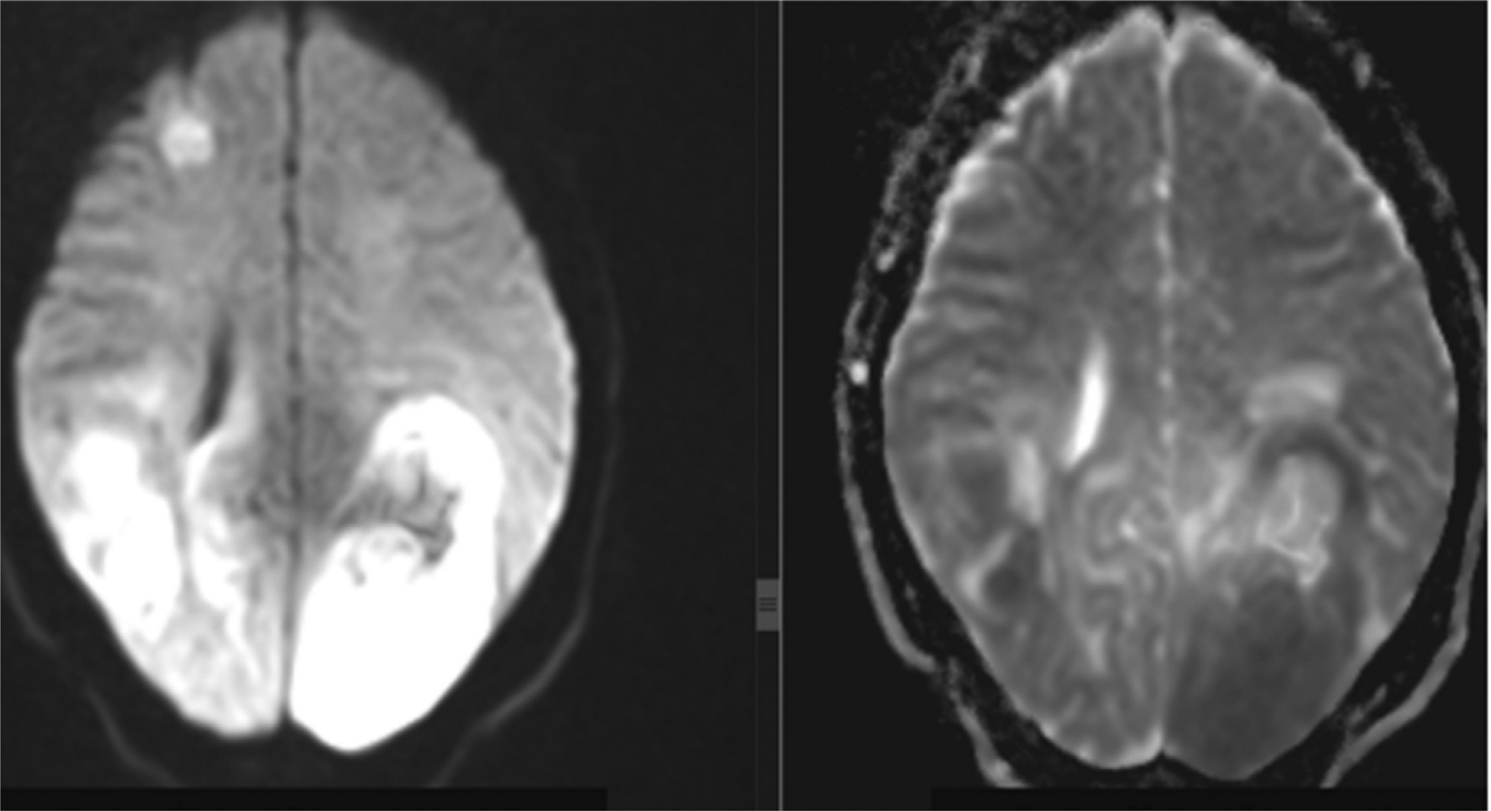
From left to right, the images show DWI and ADC maps. These maps reveal significant diffusion restriction in the lesions.

**Fig. 3:**
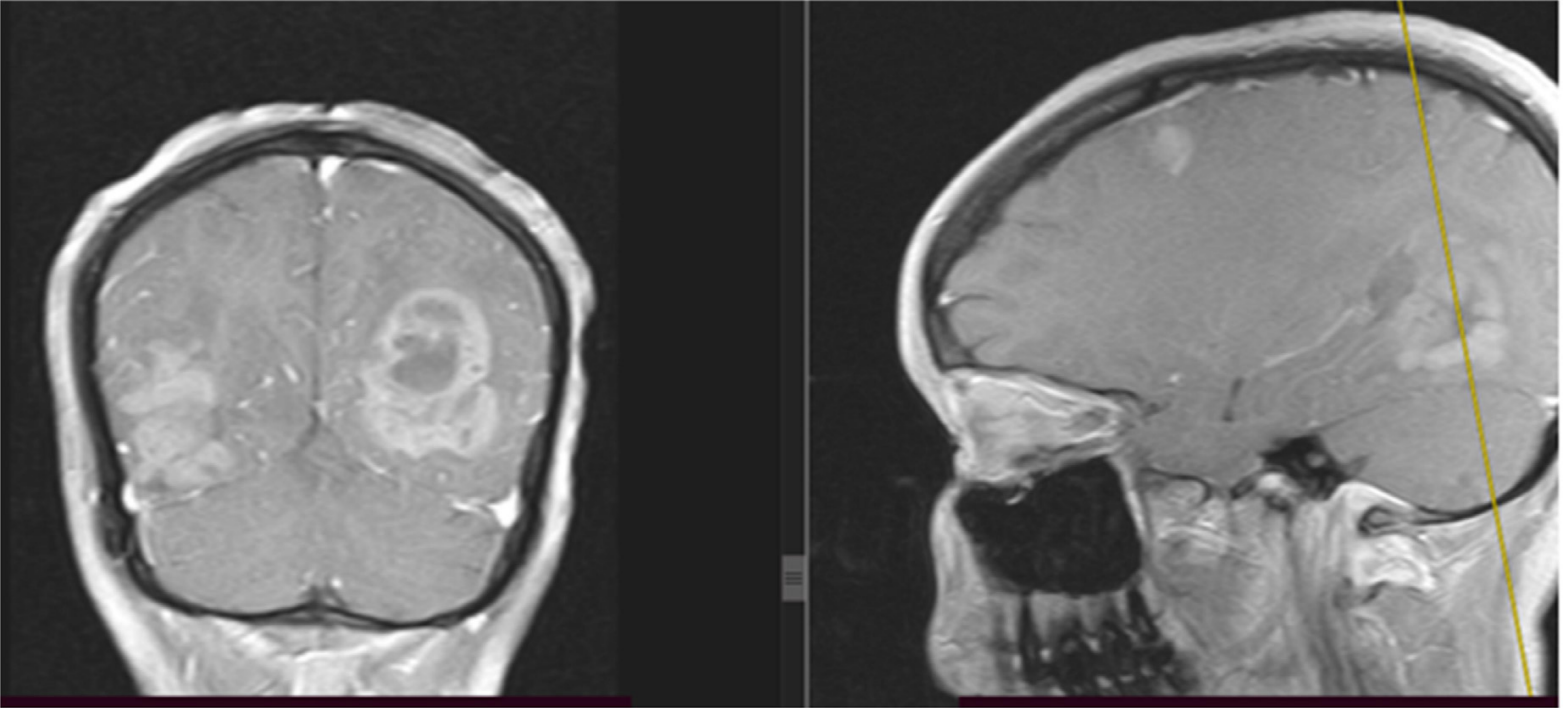
There are multiple avidly enhancing masses involving the bilateral occipitoparietal lobes, temporal lobes, and parietal lobes. Some nonenhancing components are also present.

Laboratory workup revealed no abnormalities, including a complete blood count (WBC 9700/uL [4500-11000/uL], hemoglobin 13.9 g/dL [12-15 g/dL], platelets 279,000/uL [150,000-400,000/uL]), urine beta-hCG test, liver enzymes, renal function tests, and HIV test. While CT scans of the chest and abdomen didn't show any abnormalities, the brain MRI revealed multiple bilateral cortical and subcortical masses deep in the periventricular region. These masses appeared T1-hypointense, T2-heterogeneously hyperintense, and exhibited diffusion restriction with surrounding perilesional edema (Fig. 1, Fig 2, Fig. 3).

**Fig. 4:**
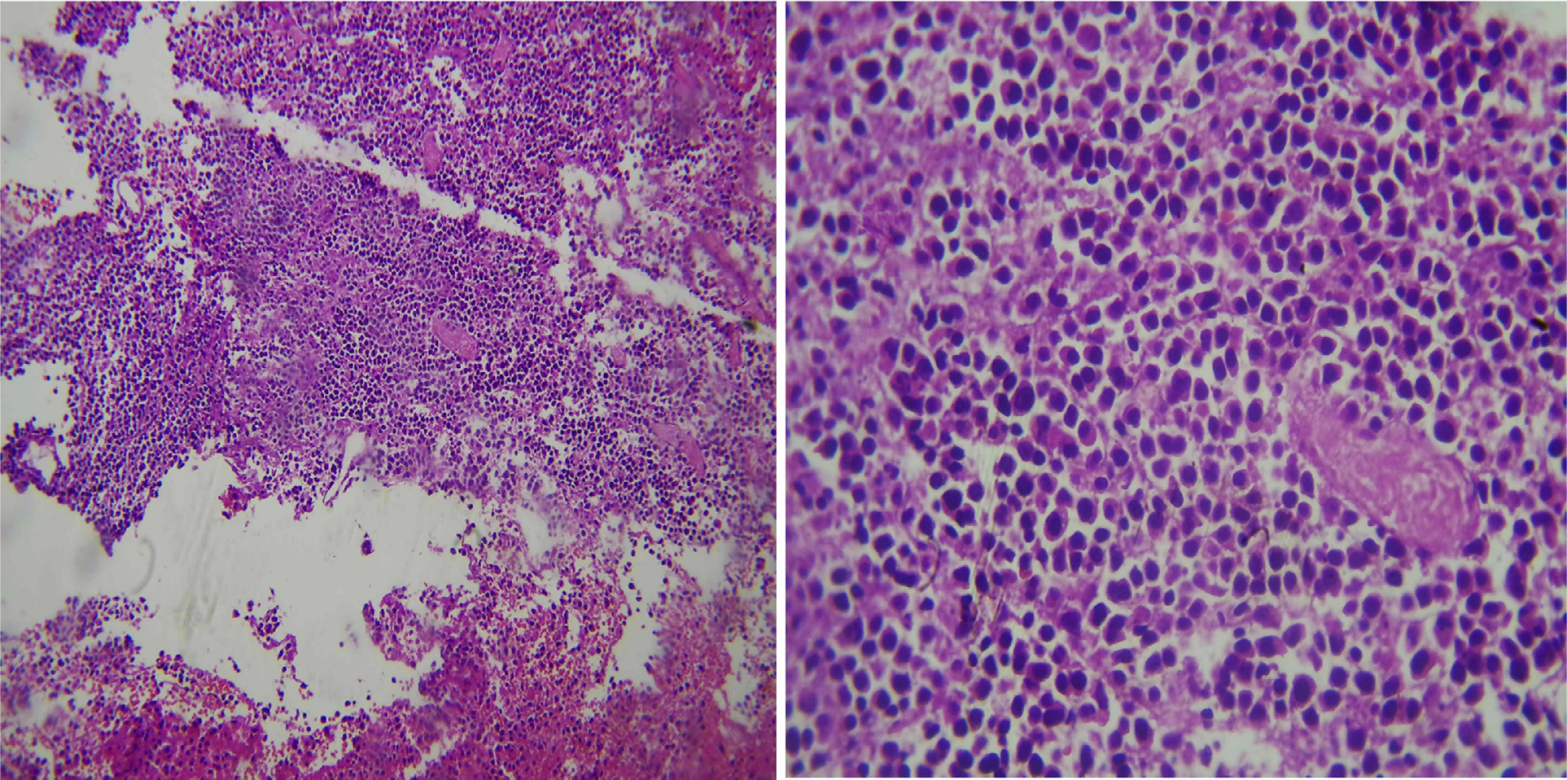
Proliferation of monotonous enlarged lymphoid cells against a fibrillary background. (A) 10× magnification, (B) 40× magnification (Fig. 5b).

**Fig. 5:**
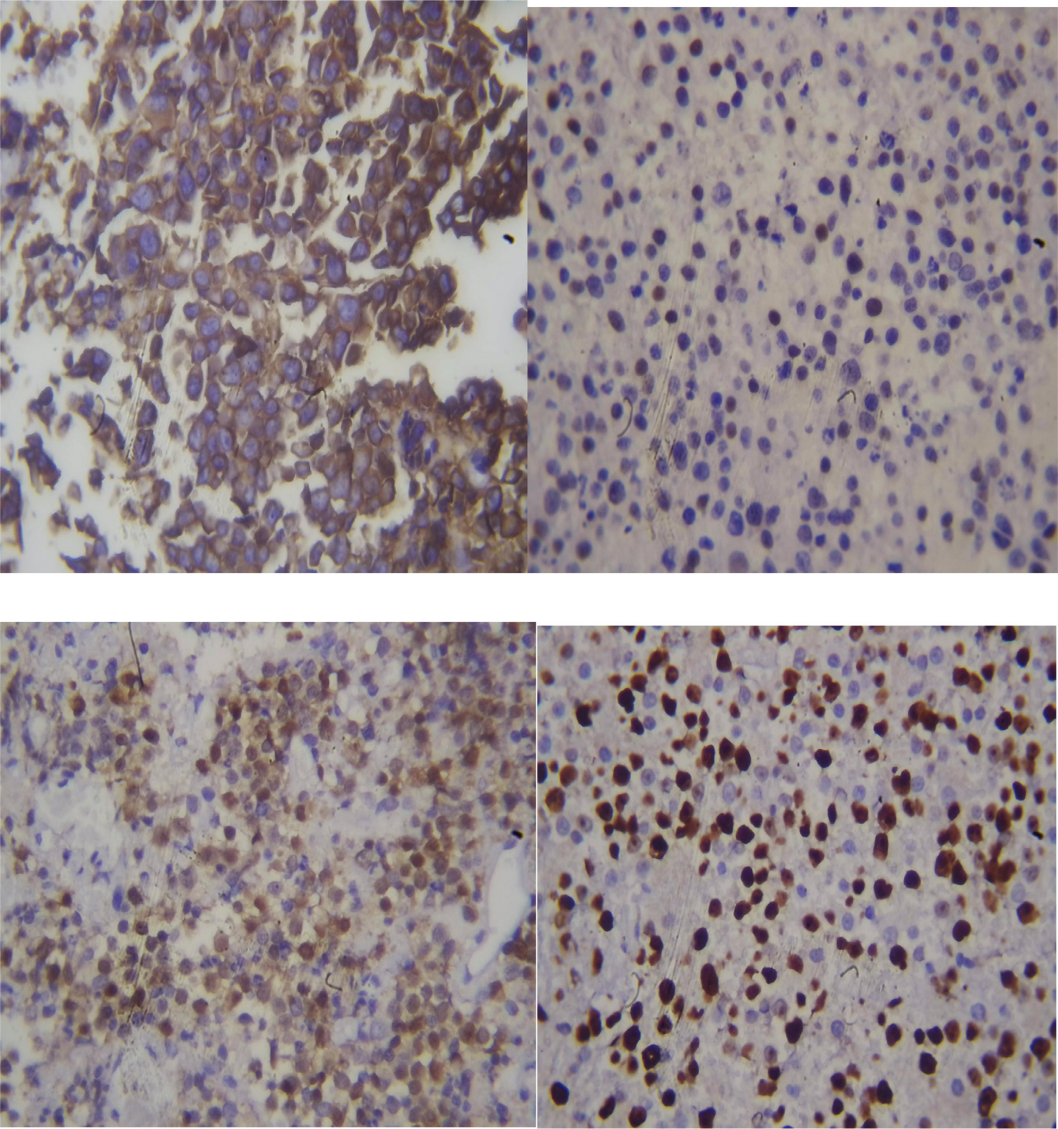
(A) CD-20 showed diffusely positive tumor cells. (B) BCL-6 was positive in 75% of cells. (C) MUM-1 was positive in 90% of cells. (D) Ki-67 proliferation index was 75%.

Left occipital craniotomy and tumor resection was done and histopathological examination of the biopsy specimen revealed moderate to large cells arranged in a diffuse growth pattern. These cells possessed prominent nucleoli (Fig. 4). Immunohistochemical analysis further characterized the tumor as diffuse large B-cell lymphoma (DLBCL), nongerminal center B-cell (non-GCB) type. The Ki-67 proliferation index was high at 75%. Additionally, the tumor cells expressed CD20, BCL6, and MUM1, while lacking expression of CD5 and CD10 (Fig. 5).

The diagnosis of PCNSL was confirmed and decided to start treatment with high-dose methotrexate-based chemotherapy. However, the patient declined this treatment and opted to leave the hospital against medical advice. The delay in diagnosis and prolonged hospitalization contributed to her decision. This prolonged hospitalization was due to the necessity of an open biopsy for tissue collection, a subsequent surgical site infection, and the unavailability of on-site immunohistochemical analysis.

## Discussion

PCNSL can be challenging to diagnose because it can mimic other conditions in the brain. These include high-grade gliomas, metastases, and infectious or granulomatous diseases. Clinical symptoms and MRI findings can be similar. Therefore, advanced MRI techniques such as MR spectroscopy may be needed to aid in the diagnosis of PCNSL [9]. In our case, MRI features of heterogeneous enhancement and a patient profile of young age, female sex, and immunocompetent further complicated the diagnostic process, as these factors are not typical for PCNSL.

The median age of PCNSL incidence in immunocompetent patients is 53-57 years and 31-35 years in immunocompromised patients. There is no sex difference in immunocompetent, but it is more common in males in immunocompromised [5,10]. In comparison, our patient is only 35 years old and immunocompetent. Though advanced age and immunosuppression are the most described risk factors, autoimmune diseases are also risk factors in immunocompetent individuals. The relative incidence of autoimmune disease is 5%-10% in PCNSL [11]. The most common clinical presentation of PCNSL in immunocompetent patients is focal neurologic deficit, followed by neuropsychiatric symptoms, signs of raised intracranial pressure like headache, nausea, vomiting, seizures, and ocular symptoms [10]. Our patient presented with headache, seizure, difficulty maintaining balance, and blurring of vision.

The most common MRI finding was a solitary brain lesion in 65% of the cases. The most frequent locations were the cerebral hemispheres, the basal ganglia, and the corpus callosum [12]. In immunocompromised patients with PCNSL, it tends to be multiple, more hemorrhagic, and peripherally enhanced than immunocompetent [5]. Our patient has multifocal lesions with bilateral cerebral cortical and subcortical involvement. And, the presence of heterogeneous enhancement was against the diagnosis of PCNSL.

DLBCL comprises 90%-95% of all PCNSL. It expresses B-cell markers, particularly CD19, CD20, CD22, CD79a, and PAX5. Bcl-6 and MUM1 are also expressed in the majority of primary CNS DLBCL. However, CD10 expression is less common, only detected in less than 10% of patients. DLBCL can be germinal center B-cell-like (GCB) and activated B-cell-like (ABC) subtypes based on cell origin. The non-GCB is the predominant subtype [13] . The patient's brain biopsy showed that DLBCL, non-GCB type, had a Ki67 proliferative index of 75%, and the cells expressed CD 20, bcl-6, and MUM 1 but not CD5 and CD10.

A multicenter Spanish study revealed a median diagnosis delay of 47 days for PCNSL in immunocompetent patients. This delay was attributed to poor performance status and the use of corticosteroids. However, discontinuation of corticosteroids before surgery did not reduce the risk of needing a repeat biopsy [14]. Another study reported median diagnosis times: 70 days for immunocompromised and 75 days for immunocompetent patients. It showed the potential for diagnostic delays across patient populations [15]. Patients who did not receive treatment earlier have a higher risk of short survival [14]. Our patient had four months of delay to diagnosis. The first delay was due to late presentation, unavailability of onsite immunohistochemistry, and surgical site infection further delayed the diagnosis.

Though there has been advancement in the management of PCNSL in the past 3 decades, the outcome is still unsatisfactory.The current guidelines recommend induction with a high dose of methotrexate-based chemotherapy followed by consolidation chemotherapy or radiotherapy. Emerging evidence suggests that autologous stem cell transplantation, Bruton's tyrosine kinase inhibitors, immunomodulatory drugs, immune checkpoint inhibitors, and chimeric antigen receptor T-cell therapy were effective and had tolerable side effects [[16], [17], [18]]. Older age, lower performance status, elevated serum lactate dehydrogenase level, increased protein concentration in the cerebrospinal fluid, and involvement of deep brain structures are independent predictors of poor survival outcomes in PCNSL [19]. Patients with HIV have a worse prognosis compared to immunocompetent PCNSL patients with a similar clinical profile. Good performance status and the use of HAART can improve survival in HIV-positive patients [20].

## Conclusion

Our experience with this case underscores the importance of maintaining a broad differential diagnosis for patients presenting with solitary or multiple brain lesions. PCNSL should be considered even in immunocompetent individuals. Minimally invasive biopsy techniques, whenever feasible and clinically appropriate, should be prioritized to minimize patient morbidity. Improving the accessibility of immunohistochemical analysis is crucial for establishing an accurate diagnosis and guiding optimal treatment regimens. Early diagnosis and treatment initiation remain the cornerstone of achieving favorable outcomes in PCNSL patients.

## Patient consent

Written informed consent was obtained from the patient for publication of this case report and accompanying images. A copy of the written consent is available for review by the Editor-in-Chief of this journal on request.
